# The Role of Body Fat and Fat Distribution in Hypertension Risk in Urban Black South African Women

**DOI:** 10.1371/journal.pone.0154894

**Published:** 2016-05-12

**Authors:** Cindy George, Julia H. Goedecke, Nigel J. Crowther, Nicole G. Jaff, Andre P. Kengne, Shane A. Norris, Lisa K. Micklesfield

**Affiliations:** 1 Non-Communicable Disease Research Unit, South African Medical Research Council, Parow, Cape Town, South Africa; 2 South African Medical Research Council/University of the Witwatersrand Developmental Pathways for Health Research Unit, Department of Pediatrics, Faculty of Health Sciences, University of Witwatersrand, Johannesburg, South Africa; 3 Department of Chemical Pathology, National Health Laboratory Service, University of the Witwatersrand Medical School, Johannesburg, South Africa; Leeds Beckett University, UNITED KINGDOM

## Abstract

Developing countries are disproportionately affected by hypertension, with Black women being at greater risk, possibly due to differences in body fat distribution. The objectives of this study were: (1) To examine how different measures of body composition are associated with blood pressure (BP) and incident hypertension; (2) to determine the association between baseline or change in body composition, and hypertension; and (3) to determine which body composition measure best predicts hypertension in Black South African women. The sample comprised 478 non-hypertensive women, aged 29–53 years. Body fat and BP were assessed at baseline and 8.3 years later. Body composition was assessed using dual-energy X-ray absorptiometry (DXA) (n = 273) and anthropometry. Hypertension was diagnosed based on a systolic/diastolic BP ≥140/90 mmHg, or medication use at follow-up. All body composition measures increased (p<0.0001) between baseline and follow-up. SBP and DBP increased by ≥20%, resulting in a 57.1% cumulative incidence of hypertension. Both DXA- and anthropometric-derived measures of body composition were significantly associated with BP, explaining 3–5% of the variance. Baseline BP was the most important predictor of hypertension (adjusted OR: 98–123%). Measures of central adiposity were associated with greater odds (50–65%) of hypertension than total adiposity (44–45%). Only change in anthropometric-derived central fat mass predicted hypertension (adjusted OR: 32–40%). This study highlights that body composition is not a major determinant of hypertension in the sample of black African women. DXA measures of body composition do not add to hypertension prediction beyond anthropometry, which is especially relevant for African populations globally, taking into account the severely resource limited setting found in these communities.

## Introduction

Hypertension is a global problem, currently affecting more than 1 billion individuals worldwide [1]. It is a disease that disproportionately affects low- and middle income countries, in particular sub-Saharan Africa (SSA) [2,3]. The burden of hypertension also varies across ethnic groups, with populations of African ethnicity being at higher risk than Caucasians [4]. Since, higher-than-optimal blood pressure levels have been reported to be the biggest single contributor to the global burden of cardiovascular disease (CVD) [1], the increasing prevalence of hypertension in the black population may also be driving an epidemic of CVD in this population.

Obesity, which is a well-recognized risk factor for hypertension [5,6], has become a major health problem in many developing countries [7–9], particularly among adult women [10,11]. Recent figures from the Global Burden of Disease 2013 Expert group indicate that Black South African (SA) women have the highest prevalence of overweight and obesity in sub-Saharan Africa, at 69.3% [12]. The disparity in the prevalence of hypertension between Black and White ethnic groups, with the black population being at higher risk than the white population, may be partly attributed to differences in body fatness and body fat distribution. Large differences in body fat distribution between Black and White South African women, have been reported, with Black woman having less central fat, but more peripheral fat than White women, for a given body mass index (BMI) [13,14]. To our knowledge, very few studies have monitored longitudinal changes in body composition in relation to hypertension risk [15–21], with only one conducted in a Black African adult population [19]. Two additional studies were in a sample of black African youths [15,18].

Historically, body composition has been measured using basic anthropometry (BMI, waist and hip circumferences), however dual-energy X-ray absorptiometry (DXA) technology is now available in which total body composition and body fat distribution can be measured with a high degree of accuracy and objectivity [22,23]. Indeed, DXA technology is not always accessible in developing countries and therefore it is important to evaluate whether the more accurate measurements of DXA have any advantages over basic anthropometric measures in predicting hypertension, especially in the light of the increased prevalence of hypertension amongst the younger age groups [24].Therefore, in a prospective study of Black SA women, the aims of this study were: (1) To examine how different measures of body fat and fat distribution are associated with blood pressure (BP) and incident hypertension; (2) to determine the association between baseline- or change in body fat and fat distribution and hypertension; and (3) to determine which body composition measure best predicts hypertension in Black South African women.

## Materials and Methods

### Study design, setting and participants

**Fig 1:**
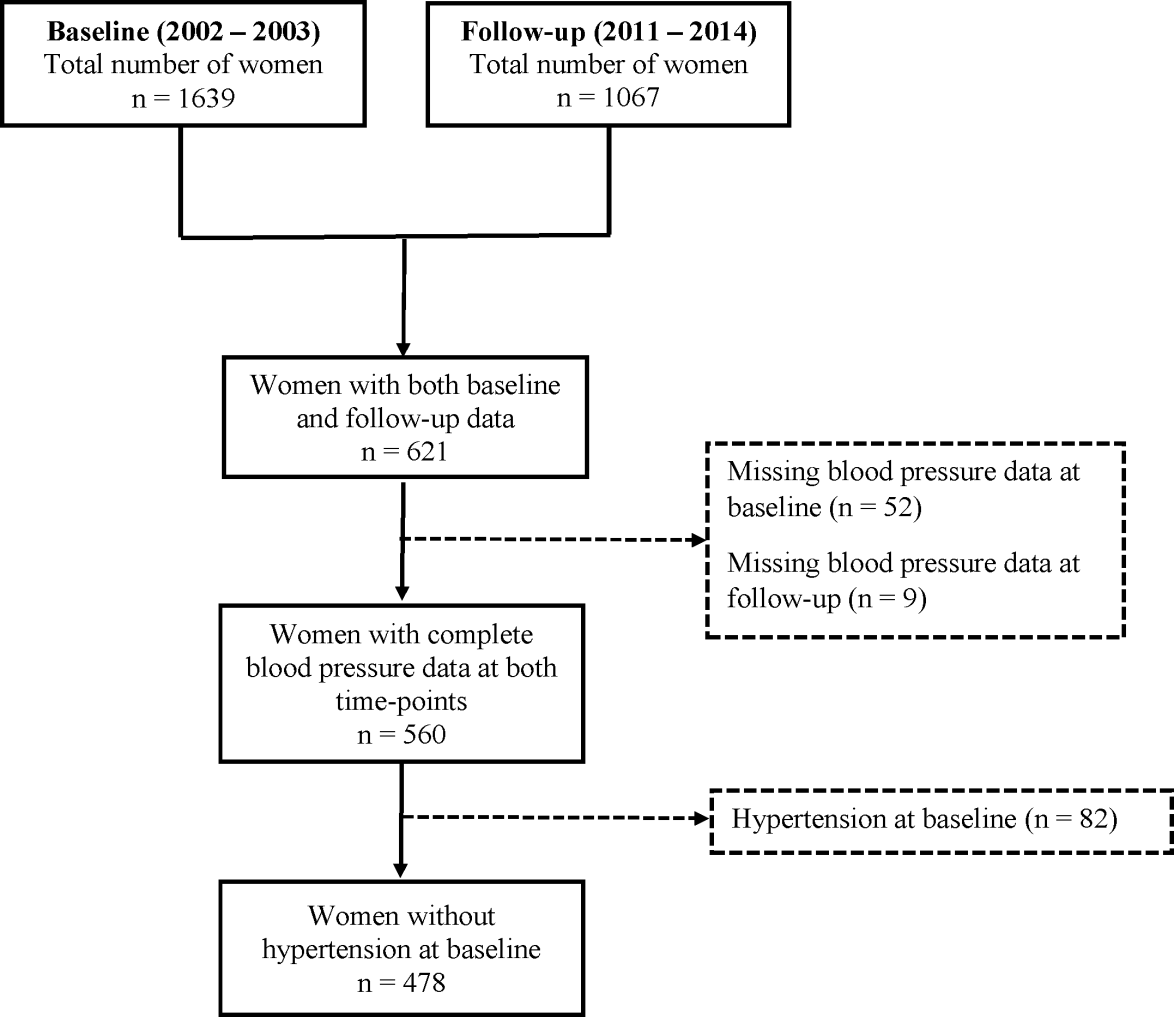
Sample selection flow chart.

This prospective study, consisting of two waves of data collection, took place at the Medical Research Council (MRC)/University of the Witwatersrand (WITS) Developmental Pathways for Health Research Unit (DPHRU) at the University of the Witwatersrand. The first wave of data collection (baseline data) took place between 2002 and 2003 and the follow-up period was between 2011 and 2014, with an average follow up period of 8.3±1.1 years (S1 Dataset). The current study consisted of Black South African female caregivers of the Birth to Twenty cohort, the largest longitudinal birth cohort study of childhood development and health in Africa to date. We excluded all participants with missing data on blood pressure at either of the time-points, with high blood pressure [systolic blood pressure (SBP) ≥140 mmHg and/or diastolic blood pressure (DBP) ≥90 mmHg] or using anti-hypertensive medication at baseline, HIV/AIDS-infected or using anti-retroviral medication, currently pregnant and lactating women. This resulted in an analytic sub-sample of 478 women aged between 29–53 years (Fig 1). The study was conducted in accordance with the guidelines of the Declaration of Helsinki and was approved by The Human Research Ethics Committee (Medical) of the University of the Witwatersrand (M010556 and M090620). All participants gave written informed consent to participate in our study after the procedures and risks were explained to them.

### Data source and measurements

During baseline recruitment, questionnaires were used to assess possible confounders including age, socio-economic status (SES), tobacco use, physical activity, personal history of hypertension and treatment, and family history of hypertension. The SES was based on household amenities [25]. Participants were classified as non-smokers, if they had never smoked, former smokers if they were smokers but stopped smoking prior to the time of the interview, and current smokers if they smoked more than 1 cigarette per day at the time of the interview. Self-reported physical activity was determined using the Global Physical Activity Questionnaire (GPAQ) [26]. Potential anthropometric-derived predictors of hypertension, including waist circumference (WC; measured at the level of the umbilicus), were measured using standard procedures previously used in research studies on similar samples. In addition, waist-to-height ratio (WHtR) [27] and body mass index (BMI) were calculated for all participants. Body weight and height were measured to the nearest 0.1 kg and 0.1 cm, respectively, by using standardized equipment and procedures. Potential DXA-derived predictors, including whole body, trunk, arm and leg fat mass (expressed as % fat mass and kg) were measured, as previously described [28], using dual-energy x-ray absorptiometry (Hologic Discovery-A (S/N 83145), Bedford, MA, USA, software version 12.5:7). The coefficient of variation (CV) was less than 0.5% for all parameters.

During follow-up, the same anthropometric and DXA measures were recorded and a similar questionnaire was used to assess tobacco use, physical activity, personal history of hypertension and treatment, and family history of hypertension. Information regarding SES was not included in the follow-up questionnaire. Questions related to menopause status and contraceptive use were included in the follow-up questionnaire. The Stages of Reproductive Ageing Workshop + 10 (STRAW+10) criteria [28,29] were used to classify menopausal status.

The outcome variables of our study were absolute blood pressure at baseline and follow-up, the change in blood pressure over time, and incident hypertension in this population. Blood pressure measurements were taken in a seated position after 30 minutes of seated rest. The measurement conditions (e.g. time of day) and environment were similar for baseline and follow-up measures. The SBP and DBP were recorded twice on the right arm using a standard mercury sphygmomanometer and appropriately sized cuff. The average of the two measures was used to determine blood pressure levels. Pulse pressure (PP) was determined by subtracting the DBP from the SBP and the mean arterial pressure (MAP) was calculated based on the formula: [DBP + PP/3]. Participants were classified as being hypertensive if they had a SBP ≥ 140 mmHg and/or DBP ≥ 90 mmHg [1], or were on hypertensive medication at the time of the interview. Participants with one or both biological parents who previously or currently had hypertension, were classified as having a family history of hypertension.

From the total sample of 478 participants that underwent anthropometric measurements, only 273 underwent DXA analysis at both time points. The participants who had DXA measures were similar in age, BMI, WC and blood pressure to those who did not have DXA measures (data not shown).

### Statistical analysis

Normally distributed data are presented as mean ± standard deviation (SD) and skewed variables as median and 25^th^-75^th^ percentiles. Change in body composition and blood pressure between baseline and follow-up evaluations were assessed using paired-sample Student-t tests or Wilcoxon-rank test. Pearson’s correlation coefficients were used to assess the relationships between body composition measures and blood pressure variables. Based on the results of these associations, robust multiple linear regression models were used to assess the independent effects of body fat and fat distribution, as well the change in these variables, on blood pressure levels, while adjusting for age at baseline. Multivariable logistic regression models were used to determine potential anthropometric- and DXA-derived predictors of hypertension, while adjusting for baseline age and baseline systolic blood pressure. In separate linear and logistic regression analysis (included in S1–S4 Tables), physical activity, family history of hypertension and smoking were included as possible covariates (n = 189). In addition, data was stratified by menopausal status at follow-up and all above-mentioned linear and logistic regression analysis reanalysed (data not shown). Standardized body composition measures were used in order to compare the effect sizes of the association directly with each other. Odds ratios (OR’s) and 95% confidence intervals for each SD increase in the body composition measure are presented. Area under the Receiver Operator Characteristic (ROC) curve (AUC) was used to determine the discriminatory power of a model, and to describe the probability that a test would correctly identify participants with hypertension.

In the sub-group of participants with data available on all anthropometric- and DXA-derived measures (n = 273), anthropometric-derived measures of total body fat (BMI) and central body fat (WC and WHtR) were compared to DXA-derived measures of total body fat (fat mass) and central body fat (trunk fat mass) as predictors of hypertension. AUC was used to determine the discriminatory power of these models. Statistical significance was based on a p<0.05. All analyses were performed using STATA version 13 (Statcorp, College Station, TX).

## Results

### Baseline characteristics of the participants and changes during follow-up

Table 1 summarizes the participants’ characteristics at baseline and follow-up. At baseline, the median (25^th^-75^th^ percentiles) age of the sample population was 40 (36–45) years, and the mean (±SD) follow-up period was 8.3±1.1 years (range: 7–12 years). At baseline, 38.5% of the participants had access to running water and 47.5% had flushing toilets inside their homes. No SES data was available at follow-up. Few women smoked at baseline (4.1%), but this more than doubled at follow up (9.9%, p<0.0001). Information related to menopausal status and contraceptive use was not included in the questionnaire at baseline, however at follow-up, 31% of the women were pre-menopausal, 23.1% were peri-menopausal and 45.9% were post-menopausal. Fourteen percent of women were using hormonal contraceptives.

**Table 1:** Subject characteristics at baseline and follow-up.

Variables	n	Baseline	Follow-up	Absolute change	Relative change (%)	Unadjusted p-value
Age (years)	478	40 (36–45)	48.5 (44–53)			
**Anthropometry**						
Height (m)	478	1.6 ± 0.1	1.6 ± 0.1	Not applicable	Not applicable	
Weight (kg)	478	75.1 ± 15.6	80.6 ± 17.6	5.5 ± 9.3	7.8 ± 13.4	< 0.0001
Waist-circumference (cm)	475	86.0 (77.0–94.0)	97.1 (88.5–106.0)	11.0 (5.0–17.0)	12.7 (5.8–20.0)	< 0.0001
WHtR	475	0.77 ± 0.08	0.83 ± 0.08	0.07 ± 0.07	9.21 ± 10.16	< 0.0001
BMI (kg/m^2^)	478	29.9 ± 6.0	32.3 ± 6.8	2.4 ± 3.7	8.6 ± 13.3	< 0.0001
**DXA-derived body composition**						
Fat mass (kg)	273	28.9 ± 9.3	31.7 ± 10.0	2.8 ± 6.2	12.8 ± 25.9	< 0.0001
Fat mass (%)	273	42.3 (37.1–46.1)	42.4 (37.6–46.0)	0.3 (-2.3–2.7)	0.7 (-5.4–7.4)	0.2981
Trunk FM (kg)	273	12.8 ± 4.8	14.1 ± 5.0	1.3 ± 3.2	15.2 ± 33.1	< 0.0001
Trunk FM (% FM)	273	43.7 ± 5.8	44.2 ± 6.1	0.4 ± 3.4	1.3 ± 8.2	0.0287
Arm FM (kg)	273	3.3 ± 1.3	3.6 ± 1.2	0.3 ± 0.9	17.2 ± 34.5	< 0.0001
Arm FM (% FM)	273	11.3 ± 1.8	11.5 ± 1.7	0.2 ± 1.4	3.1 ± 13.2	0.0071
Leg FM (kg)	273	12.8 ± 4.0	14.0 ± 4.7	1.2 ± 2.5	10.6 ± 21.3	< 0.0001
Leg FM (% FM)	273	45.0 ± 6.8	44.3 ± 6.5	-0.7 ± 3.4	-1.2 ± 7.4	0.0012
**Blood pressure**						
Systolic BP (mmHg)	478	109 .7 ± 12.4	131.5 ± 18.8	21.8 ± 18.0	20.7 ± 17.2	< 0.0001
Diastolic BP (mmHg)	478	72.3 ± 8.8	86.5 ± 11.3	14.2 ± 10.7	20.5 ± 16.2	< 0.0001
Pulse pressure (mmHg)	478	37.4 ± 8.2	45.0 ± 12.5	7.7 ± 12.6	24.3 ± 37.3	< 0.0001
MAP (mmHg)	478	157.9 ± 17.2	189.1 ± 25.1	31.3 ± 23.7	20.5 ± 15.9	< 0.0001

Data presented as means ± SD or median (25^th^-75^th^ percentiles) depending on the distribution of the data. WHtR, waist-to-height ratio; BMI, body mass index; FM, fat mass; BP, blood pressure; MAP, mean arterial pressure.

### Baseline body composition and fat distribution and changes during follow-up

All measures of body composition increased significantly from baseline to follow-up (all p<0.0001, Table 1). On average, BMI increased by 8.6%, WC by 12.5%, HC by 3.8%, and total body fat mass increased by 12.8%. When examining changes in regional fat distribution [expressed as a percentage of total fat mass (%FM)], there was a significant increase in trunk FM and arm FM, and a significant decrease in leg FM.

### Baseline blood pressure, change in blood pressure and incident hypertension

At baseline, 41.8% of the women had a family history of hypertension, which increased to 48.1% at follow-up (p<0.0001). Both SBP and DBP increased significantly over the follow-up period, with SBP increasing by 20.7% and DBP increasing by 20.5% (both p<0.0001, Table 1). Similarly, the derivatives of these blood pressure measures also increased significantly over the follow-up period, with PP increasing by 24.3% and MAP increasing by 20.5% (both p<0.0001). All participants were free of hypertension at baseline, however at follow-up, more than half of the participants presented with hypertension, culminating in a 57.1% hypertension incidence.

### Associations between anthropometric-derived measures of body composition, and blood pressure at follow up

The age-adjusted associations between baseline and change in anthropometric-derived measures of body composition, and blood pressure at follow-up, are presented in Table 2. Baseline and change in anthropometric-derived measures of central fat, namely WC and WHtR, accounted for 4–5% of the variance in SBP, DBP and MAP, after adjusting for age. Baseline and change in BMI, a measure of total body fat, was only significantly associated with DBP, and accounted for 3% of the variance in DBP. Similarly, baseline and change in HC was significantly associated with DBP, and accounted for 3% of the variance in DBP. However, only change in HC was associated with MAP, accounting for 2% of the variance in this measure. No baseline or change in anthropometric measures of body composition correlated with PP.

**Table 2:** Regression coefficients from robust multiple linear models for the prediction of blood pressure at follow-up by anthropometric-derived measures, accounting for potential effects of age.

Anthropometric		SBP				DBP				MAP				PP			
measures		β	SE	*p*	R^2^	β	SE	*p*	R^2^	β	SE	*p*	R^2^	β	SE	*p*	R^2^
**BMI**	**Baseline**	0.08	0.06	0.207	0.03	0.13	0.05	0.010	0.03	0.04	0.02	0.090	0.03	0.04	0.17	0.793	0.04
	**Change**	0.07	0.05	0.227		0.14	0.05	0.006		0.04	0.02	0.083		-0.05	0.14	0.742	
**WC**	**Baseline**	0.12	0.06	0.055	0.04	0.16	0.05	0.003	0.05	0.06	0.03	0.020	0.04	0.09	0.15	0.562	0.04
	**Change**	0.12	0.05	0.016		0.21	0.05	< 0.0001		0.07	0.02	0.002		0.02	0.13	0.909	
**HC**	**Baseline**	0.04	0.06	0.425	0.03	0.11	0.05	0.034	0.03	0.03	0.02	0.228	0.02	-0.02	0.14	0.873	0.04
	**Change**	0.08	0.05	0.110		0.14	0.06	0.010		0.05	0.02	0.046		0.01	0.11	0.912	
**WHtR**	**Baseline**	0.14	0.06	0.021	0.04	0.17	0.05	0.002	0.05	0.07	0.03	0.007	0.05	0.14	0.15	0.363	0.04
	**Change**	0.13	0.05	0.013		0.21	0.05	< 0.0001		0.07	0.02	0.001		0.01	0.12	0.920	

Each model includes baseline body composition measures, the change in the body composition measures and age as predictor variables and blood pressure measures at follow-up as the outcome variables (n = 473). Data are presented as β-coefficients, standard error (SE) and p-values (*p*) for each body fat and fat distribution variable, as well as R^2^ for each model. ‘Baseline’ represents the baseline body fat and fat distribution variable and ‘Change’ represents the change in body fat and fat distribution variable; SBP, systolic blood pressure; DBP, diastolic blood pressure; MAP, mean arterial blood pressure; PP, pulse pressure; BMI, body mass index; WC, waist-circumference; HC, hip-circumference, WHtR, waist-to-height-ratio.

### Associations between DXA-derived measures of body composition, and blood pressure at follow up

The age-adjusted associations between baseline and change in DXA-derived measures of body composition, and blood pressure at follow-up, are presented in Table 3. Both the baseline and change in the DXA-derived measure of central fat, namely trunk FM, was significantly associated with DBP and MAP, accounting for 3–5% of the variance in these BP measures. In contrast, baseline and change in total fat mass was only associated with DBP, accounting for 4% of the variance. Both baseline and change in arm FM were significantly associated with DBP, accounting for 3% of the variance in DBP. Leg FM was not associated with any blood pressure measures. None of the DXA-derived measures were significantly associated with SBP or PP.

**Table 3:** Regression coefficients from multiple robust linear models for the prediction of blood pressure at follow-up by DXA-derived measures, accounting for potential effects of age.

DXA-derived		SBP				DBP				MAP				PP			
measures		β	SE	*p*	R^2^	β	SE	*p*	R^2^	β	SE	*p*	R^2^	β	SE	*p*	R^2^
**Fat mass**	**Baseline**	0.11	0.08	0.168	0.02	0.15	0.07	0.028	0.04	0.06	0.03	0.096	0.02	0.10	0.20	0.599	0.02
	**Change**	0.07	0.07	0.310		0.16	0.06	0.011		0.04	0.03	0.141		-0.03	0.18	0.861	
**Trunk FM**	**Baseline**	0.16	0.09	0.060	0.03	0.18	0.07	0.011	0.05	0.09	0.04	0.032	0.03	0.18	0.20	0.360	0.03
	**Change**	0.11	0.07	0.134		0.19	0.06	0.003		0.06	0.03	0.045		0.00	0.18	0.993	
**Arm FM**	**Baseline**	0.07	0.10	0.497	0.01	0.16	0.08	0.035	0.03	0.04	0.04	0.284	0.01	-0.05	0.24	0.85	0.02
	**Change**	0.04	0.07	0.571		0.15	0.06	0.021		0.03	0.03	0.284		-0.11	0.20	0.577	
**Leg FM**	**Baseline**	0.05	0.07	0.486	0.01	0.08	0.06	0.218	0.02	0.03	0.03	0.382	0.01	0.04	0.18	0.820	0.02
	**Change**	0.04	0.07	0.613		0.10	0.06	0.122		0.02	0.03	0.430		-0.03	0.17	0.853	

Each model includes baseline body fat and fat distribution measures, the change in the body fat and fat distribution measures and age as predictor variables and blood pressure measures at follow-up as the outcome variables (n = 273). Data presented as β-coefficients, standard error (SE) and p-values (*p*) for each body fat and fat distribution variable, as well as R^2^ for each model. ‘Baseline’ represents baseline body fat and fat distribution variable and ‘Change’ represents change in body fat and fat distribution variable; SBP, systolic blood pressure; DBP, diastolic blood pressure; MAP, mean arterial blood pressure; PP, pulse pressure; FM, fat mass.

### Predictors of incident hypertension

The odds of incident hypertension determined by baseline and change in anthropometric- and DXA-derived body composition measures, adjusting for age and blood pressure at baseline, are shown in Tables 4 and 5, respectively. For every one SD increase in baseline BMI, WC, HC and WHtR, the odds of developing hypertension increased by 33–65%, with the baseline WHtR having the highest odds compared to the other anthropometric-derived measures (1.65 [1.32–2.08], p<0.0001). In addition, for every one SD increase in the change in WC, HC and WHtR over the follow-up period, the odds of developing hypertension increased by 32–40%. When examining the DXA-derived predictors of hypertension, baseline fat mass, trunk FM and arm FM, rather than the change in these variables, were associated with increased odds of developing hypertension, ranging from 44–62%. There was no significant difference in the AUC between the models including either anthropometric or DXA-derived measures of body composition, suggesting that all measures of body composition similarly predicted incident hypertension.

**Table 4:** Anthropometric-derived measures of body composition as predictors of hypertension at follow-up (n = 473).

	Odds ratio	95% CI	p	AUC
**BMI**				0.76
Age	1.54	1.25–1.90	< 0.0001	
Baseline BP	2.19	1.75–2.75	< 0.0001	
Baseline BMI	1.45	1.17–1.79	0.001	
Δ BMI	1.22	1.00–1.50	0.057	
**WC**				0.76
Age	1.49	1.20–1.83	< 0.0001	
Baseline BP	2.21	1.76–2.77	< 0.0001	
Baseline WC	1.55	1.24–1.95	< 0.0001	
Δ WC	1.32	1.06–1.63	0.012	
**HC**				0.76
Age	1.54	1.25–1.89	< 0.0001	
Baseline BP	2.21	1.76–2.76	< 0.0001	
Baseline HC	1.33	1.07–1.64	0.010	
Δ HC	1.40	1.12–1.74	0.003	
**WHtR**				0.77
Age	1.48	1.20–1.83	< 0.0001	
Baseline BP	2.23	1.77–2.80	< 0.0001	
Baseline WHR	1.65	1.31–2.08	< 0.0001	
Δ WHtR	1.35	1.08–1.67	0.007	

Data presented as odds ratio, 95% confidence interval (CI), area under ROC curve (AUC). BP, blood pressure; Δ, change in body composition; BMI, body mass index; WC, waist circumference; HC, hip circumference; WHtR, waist-to-height ratio.

**Table 5:** DXA-derived measures of body composition as predictors of hypertension at follow-up (n = 273).

	Odds ratio	95% CI	p	AUC
**Fat mass**				0.74
Age	1.43	1.09–1.88	0.010	
Baseline BP	2.01	1.51–2.66	< 0.0001	
Baseline FM	1.44	1.06–1.96	0.019	
Δ FM	1.17	0.88–1.55	0.287	
**Trunk fat mass**				0.74
Age	1.42	1.08–1.86	0.013	
Baseline BP	2.02	1.52–2.69	< 0.0001	
Baseline trunk FM	1.50	1.10–2.06	0.011	
Δ trunk FM	1.20	0.90–1.60	0.226	
**Arm fat mass**				0.74
Age	1.40	1.06–1.84	0.016	
Baseline BP	1.98	1.50–2.63	< 0.0001	
Baseline arm FM	1.62	1.13–2.32	0.009	
Δ arm FM	1.18	0.86–1.62	0.314	
**Leg fat mass**				0.73
Age	1.46	1.11–1.91	0.006	
Baseline BP	2.02	1.52–2.67	< 0.0001	
Baseline leg FM	1.24	0.94–1.63	0.130	
Δ leg FM	1.08	0.83–1.41	0.552	

Data presented as odds ratio, 95% confidence interval (CI), area under ROC curve (AUC). BP, blood pressure; Δ, change in body composition; FM, fat mass.

### DXA-derived measures versus anthropometric-derived measures of body composition as predictors of hypertension

**Fig 2:**
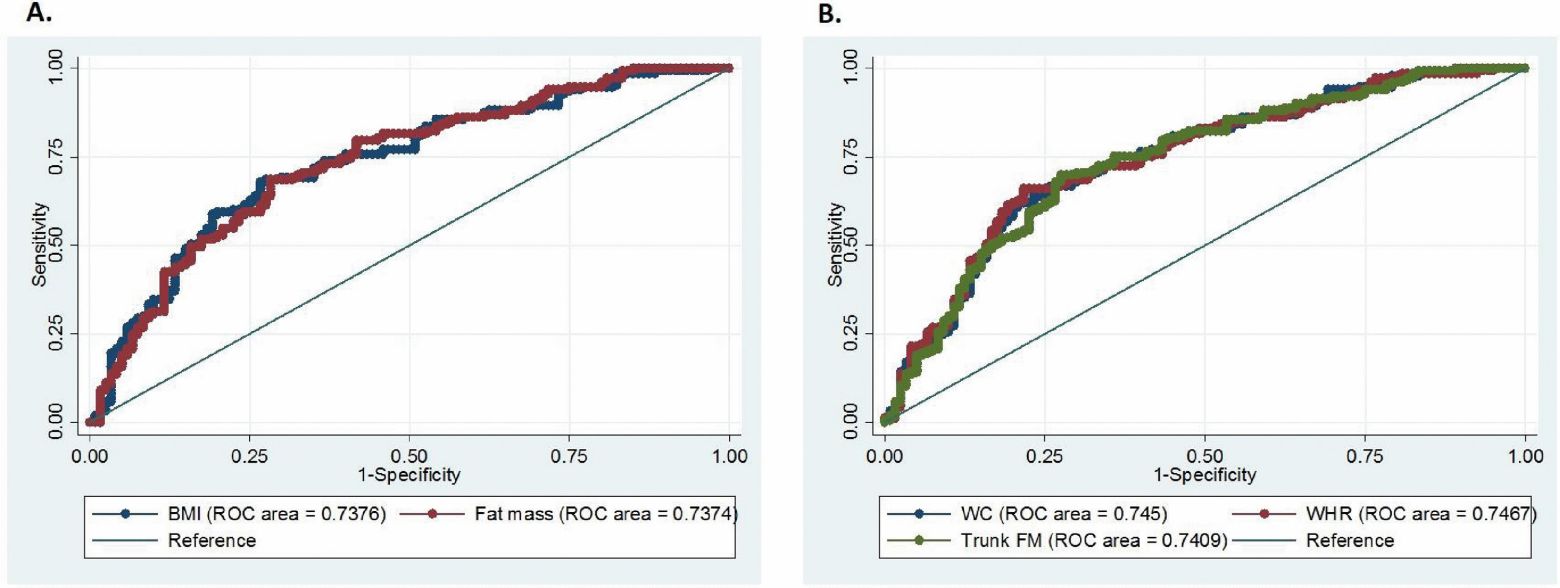
**Anthropometric-derived measure vs. DXA-derived measure of (A) total adiposity and (B) central adiposity as predictors of hypertension.** There are no significant differences in areas under curve (AUC) for the different body composition measures [(A.) p = 0.9785 and (B.) p = 0.7965] (n = 273). ROC, receiver operating characteristic (ROC); WC, waist circumference; WHtR, waist-to-height ratio; FM, fat mass.

When comparing anthropometric- to DXA-derived measures of total body fat mass (BMI vs. fat mass) in predicting hypertension, no significant difference was found between the models (AUC: 0.738 vs. 0.737, respectively, Fig 2A). Similarly, when comparing anthropometric- to DXA-derived measures of central fat (WC and WHtR vs. trunk FM), no significant differences were found between the models (AUC: 0.745 and 0.747 vs. 0.741, respectively Fig 2B).

## Discussion

In this study of middle-aged black SA women, we have shown a hypertension incidence rate of 57% over an 8 year period. Although baseline BP was the most significant predictor of incident hypertension, central measures of adiposity at baseline were also associated with greater odds of developing hypertension when compared to whole body measures of adiposity, as well as change in adiposity over time. Total body fat and fat distribution was determined using both anthropometric- and DXA-derived measures of body composition, and we showed that DXA-derived measures provided no additional benefit over anthropometry-derived measures, such as WC and WHtR, in our sub-sample of 273 participants. This is especially relevant for African populations globally, taking into account the severely resource limited setting found in these communities.

The cumulative incidence of hypertension in our study meant that three out of five women in this population developed hypertension within a period of approximately 8 years, which is 16% higher than the average for SA women, as reported by the WHO [30]. This high rate of hypertension found in our study might partially be explained by the menopausal transition. Nearly half our sample were post-menopausal at follow-up and it is well known that menopause is strongly associated with increased hypertension risk, likely due to increased activation of the renin-angiotensin system [31], the rapid drop in oestrogen [32,33], obesity and in particular the redistribution of body fat, favouring an increase in abdominal fat [34,35]. However, when data were stratified by menopausal status at follow-up, or when the interaction between menopausal status and body fat distribution on blood pressure or hypertension were explored, the interpretation of the results was not altered. The incidence and prevalence of hypertension are also much higher in Black women compared to women of other ethnic groups [36,37]. This may be, in part, attributed to the higher prevalence of obesity in this population. In SA, the estimated prevalence of obesity in Black women is 39.9%, which is higher compared to the mixed-race (34.9%) and Asian/Indian (32.4%) populations [7]. Indeed, the average BMI of the women in this study was in the obese category at baseline and increased at follow-up, with more than 50% of the participants classified as obese.

The present study also showed that measures of central adiposity, including WC, WHtR and trunk FM tended to be associated with higher odds of developing hypertension, compared to measures of total body fat such as BMI and fat mass. This is in accordance with previous cross-sectional studies in a SA population [38] and various other populations that have also reported that measures of central adiposity, rather than whole body adiposity, are better at predicting hypertension risk in women [39,40]. In addition, baseline and change in the anthropometric-derived measures of central body fat, namely WC and WHtR, predicted SBP and DBP at follow up, while the whole body measures such as BMI and fat mass were only associated with DBP. Since numerous prospective epidemiological studies have reported SBP to be a better indicator of CVD risk in adults compared to DBP [41–43], this reiterates the point that the complications of obesity are more closely related to the distribution rather than the absolute degree of adiposity.

The current study showed that even though the various anthropometric-derived and DXA-derived measures of body fat and fat distribution significantly contributed to increasing blood pressure levels and hypertension incidence, the most significant contributor to blood pressure and hypertension was baseline blood pressure levels, thus the tracking of elevated blood pressure with age. It could be argued that the weak association between body composition and blood pressure may be the effect of ethnicity. In a similar African population, Schutte et al. [44], demonstrated significantly weaker correlations between obesity measures (total body fat, abdominal obesity and peripheral fat) and systolic blood pressure when comparing African women to Caucasian women. Studies have also shown that black women have less central fat and more peripheral fat mass compared to white women [14]. In addition, for similar BMI, women of African descent have less visceral (VAT)- and more abdominal subcutaneous fat (SAT), in comparison with white women [13,45]. Larger depots of abdominal VAT, rather than SAT, are associated with a higher hypertension risk in both black and white women [46], most likely due to the inflammatory markers, adipokines and growth factors being secreted by this fat depot [47]. Thus, the lower amount of VAT in black women might have resulted in the weaker association between body fat and fat distribution in relation to hypertension risk in this group. In our study we did not measure VAT and SAT, which might be stronger predictors of hypertension than WC or trunk FM in this population. It is also possible that other factors, including genetics, the lack of physical activity, excessive smoking, alcohol use and a high sodium intake, need to be considered when predicting hypertension in this population. However, we did adjust for physical activity, smoking and family history of hypertension in the regression models (S3 and S4 Tables), but none of these variables contributed significantly to hypertension risk. This may be due to the reduced sample size after adjustment, as we only had lifestyle data on 189 participants. In addition, menopausal status was not measured at baseline and was therefore not included in the regression models. There is evidence to show that the menopause transition is associated with an increase in blood pressure which is related not only to changes in body fat mass but also to decreases in the blood androgen-to-oestrogen ratio and activation of the renin-angiotensin system [48]. These latter 2 factors were not measured over the course of the current study. Based on the age range of the women in our study, a higher percentage of the women would have been premenopausal at baseline, a significant number of whom would have transitioned to peri- and postmenopause during the follow-up period. Indeed, at follow-up, 70% of the women were in the peri- and postmenopausal phase. This might also explain the pattern of fat redistribution from the periphery (legs) to the abdomen over the 8-year follow-up period as menopause is associated with a transition from a gynoid to an android pattern of fat distribution [34,35]. However, there is still a great need for research to better understand what influences hypertension in SA women beyond body fat and fat distribution.

This study also showed that in addition to the baseline body composition measures, change in body composition independently predicted incident hypertension and blood pressure, which to our knowledge has not been reported before. However, the addition of change in body composition to the baseline body composition regression models did not significantly increase the proportion of variance that could be explained by the different body composition variables (data not shown). These results are important in the practical setting, since data on change in body composition is not always available and this study highlights the fact that baseline body composition is sufficient and is in fact a stronger predictor of hypertension.

In this study we also compared anthropometric- to DXA-derived measures of body fat and fat distribution to determine whether DXA-derived measures offer additional advantage in predicting hypertension or blood pressure. When comparing the AUC of the anthropometric- with DXA-derived measures of total and central adiposity, the indices correlated similarly. Our findings therefore suggest that, compared to basic anthropometry, DXA-derived measures of body composition do not offer an additional advantage in predicting hypertension or blood pressure in this Black SA population, however additional studies need to be conducted with other cardiovascular outcomes. Similarly, Maximova [49] showed that measures of total and central fat from DXA did not show an improved ability over BMI or WC to identify children (aged 8–10 years) with elevated SBP.

A limitation of our study was that measurements were only taken at two time points, limiting the interpretation of changes during the intervening period. Other limitations include convenience sampling, small sample size for the measured covariates, and not measuring other covariates, such as sodium intake. Additionally, baseline menopausal status was not measured. To our knowledge, this is the first longitudinal study that has examined changes in body composition and body fat distribution, examining both anthropometric-derived and DXA-derived measures of body composition, in relation to hypertension risk in a female African population. The results obtained from this study are essential to understanding the role of body fat and fat distribution in relation to hypertension risk in the Black female population. Since the high incidence of hypertension is only partially explained by body fat and fat distribution, additional studies are important to determine which factors contribute most to the high incident hypertension.

In conclusion, this study highlights the high incidence of hypertension in a black female population, which is significantly associated with baseline body fat, in particular central adiposity. This highlights the necessity to measure central adiposity when screening for hypertension risk. Our study highlights that we cannot generalize risk factors of hypertension to the same extent to all populations. Body fat and fat distribution differ between ethnic groups and this study highlights that body composition is not the major determinant of hypertension in the sample of black African women, but that more critical mechanistic work is needed to determine what is causing the significant increase in hypertension with age and menopausal transitions in the African population. Finally, DXA-derived measures of body composition do not add to hypertension prediction beyond anthropometry-derived measures. Since anthropometry is an affordable and easy measure to acquire, these findings are especially valuable taking into account the severely resource limited setting found in low and middle income countries.

## Supporting Information

S1 Dataset Data used in this study. Labels used in dataset, 03, baseline; swt, follow-up; ac, absolute change, rc, relative change; bmi, body mass index; wc, waist circumference; whr, waist-to-hip ratio; wheightr, waist-to-height ratio; msbp, mean systolic blood pressure; mdbp, mean diastolic blood pressure; pp, pulse pressure; map, mean arterial pressure; pc, percentage; hypertensive_03_bp; participants with high blood pressure at baseline; hypertensive_swt_bpmeduse; participants with high blood pressure and/or using medication to treat hypertension at follow-up; fhhypertension, family history of hypertension.
(XLSX)

S1 Table Regression coefficients for multiple robust linear models for the prediction of blood pressure at follow-up by anthropometric-derived measures, adjusted for age, baseline and change in body composition, physical activity, family history of hypertension and tobacco use. Data presented as β-coefficient, standard error (SE) and p-value. As well as R^2^ for each model. ‘Baseline’ represents the baseline body fat and fat distribution variable and ‘Δ’ represents the change in body fat and fat distribution variable; SBP, systolic blood pressure; DBP, diastolic blood pressure; MAP, mean arterial blood pressure; PP, pulse pressure; BMI, body mass index; WC, waist-circumference; HC, hip-circumference, WHtR, waist-to-height-ratio; PA, physical activity; FHH, family history of hypertension.
(PDF)

S2 Table Regression coefficients for multiple robust linear models for the prediction of blood pressure at follow-up by DXA-derived measures, adjusted for age, baseline and change in body composition, physical activity, family history of hypertension and tobacco use. Data presented as β-coefficient, standard error (SE) and p-value. As well as R^2^ for each model. ‘Baseline’ represents the baseline body fat and fat distribution variable and ‘Δ’ represents the change in body fat and fat distribution variable; SBP, systolic blood pressure; DBP, diastolic blood pressure; MAP, mean arterial blood pressure; PP, pulse pressure; FM, fat mass; PA, physical activity; FHH, family history of hypertension.
(PDF)

S3 Table Multiple logistic regression analysis of anthropometric-derived measures of body composition as predictors of hypertension, adjusted for age, baseline blood pressure, baseline and change in body composition, physical activity, family history of hypertension and tobacco use. Data presented as odds ratio, 95% confidence interval (CI), area under ROC curve (AUC). BP, blood pressure; BC, body composition; Δ BC, change in body composition; PA, physical activity; FHH, family history of hypertension.
(PDF)

S4 Table Multiple logistic regression analysis of DXA-derived measures of body composition as predictors of hypertension, adjusted for age, baseline blood pressure, baseline and change in body composition, physical activity, family history of hypertension and tobacco use. Data presented as odds ratio, 95% confidence interval (CI), area under ROC curve (AUC). BP, blood pressure; Δ, change in body composition; FM, fat mass; PA, physical activity; FHH, family history of hypertension.
(PDF)
