# Supplementary material for: The Role of Body Fat and Fat Distribution in Hypertension Risk in Urban Black South African Women
Source: PLoS One. 2016 May 12;11(5):e0154894. doi: 10.1371/journal.pone.0154894 (PMC4865112; doi:10.1371/journal.pone.0154894)
Supplement: S1 Table — Data presented as β-coefficient, standard error (SE) and p-value. As well as R2 for each model. ‘Baseline’ represents the baseline body fat and fat distribution variable and ‘Δ’ represents the change in body fat and fat distribution variable; SBP, systolic blood pressure; DBP, diastolic blood pressure; MAP, mean arterial blood pressure; PP, pulse pressure; BMI, body mass index; WC, waist-circumference; HC, hip-circumference, WHtR, waist-to-height-ratio; PA, physical activity; FHH, family history of hypertension. (PDF) [file pone.0154894.s002.pdf]

S1 Table: Regression coefficients for multiple robust linear models for the prediction of blood pressure at follow-up by anthropometric-derived measures, adjusted for age, baseline and change in body composition, physical activity, family history of hypertension and tobacco use.

Anthropometric-derived measures	SBP				DBP				MAP				PP			
	β	SE	*p*	R^2	β	SE	*p*	R^2	β	SE	*p*	R^2	β	SE	*p*	R^2
**BMI**																
Age	0.12	0.07	0.079		-0.01	0.07	0.861		0.04	0.03	0.215		0.43	0.15	0.004	
Baseline BMI	0.06	0.07	0.371		0.15	0.06	0.012		0.04	0.03	0.161		-0.07	0.16	0.652	
Δ BMI	0.01	0.06	0.903	0.02	0.09	0.06	0.097	0.03	0.02	0.03	0.573	0.02	-0.16	0.15	0.305	0.04
PA	-0.03	0.13	0.833		-0.02	0.13	0.852		-0.01	0.05	0.848		-0.10	0.29	0.722	
FHH	-0.08	0.13	0.516		-0.08	0.13	0.539		-0.04	0.05	0.507		-0.14	0.29	0.626	
Smoking	-0.34	0.31	0.268		-0.16	0.22	0.454		-0.13	0.11	0.263		-0.88	0.94	0.350	
**WC**																
Age	0.11	0.07	0.113		-0.03	0.07	0.700		0.03	0.03	0.292		0.42	0.15	0.006	
Baseline WC	0.15	0.07	0.028		0.21	0.06	0.001		0.08	0.03	0.010		0.12	0.15	0.441	
Δ WC	0.09	0.06	0.137	0.04	0.21	0.05	<0.0001	0.06	0.05	0.02	0.024	0.04	-0.10	0.14	0.467	0.04
PA	-0.03	0.13	0.798		-0.03	0.12	0.799		-0.01	0.05	0.806		-0.10	0.29	0.721	
FHH	-0.10	0.13	0.448		-0.08	0.12	0.542		-0.04	0.05	0.455		-0.19	0.29	0.528	
Smoking	-0.33	0.31	0.285		-0.21	0.21	0.318		-0.13	0.12	0.256		-0.78	0.92	0.407	
**HC**																
Age	0.12	0.07	0.086		-0.02	0.07	0.809		0.04	0.03	0.232		0.44	0.15	0.004	
Baseline HC	0.03	0.06	0.644	0.02	0.12	0.06	0.046	0.03	0.02	0.03	0.357	0.02	-0.11	0.15	0.468	0.04
Δ HC	0.06	0.06	0.378		0.12	0.07	0.080		0.03	0.03	0.228		-0.04	0.13	0.778	
PA	-0.03	0.13	0.826		-0.03	0.12	0.781		-0.01	0.05	0.822		-0.08	0.29	0.774	
FHH	-0.08	0.13	0.556		-0.06	0.13	0.617		-0.03	0.05	0.560		-0.15	0.30	0.621	

Smoking	-0.37	0.31	0.235		-0.19	0.22	0.372		-0.14	0.12	0.222		-0.92	0.95	0.332	
**WHtR**																
Age	0.11	0.07	0.119		-0.03	0.07	0.676		0.03	0.03	0.306		0.42	0.15	0.006	
Baseline WHtR	0.17	0.07	0.011		0.21	0.06	0.001		0.08	0.03	0.004		0.16	0.15	0.293	
Δ WHtR	0.09	0.06	0.117	0.04	0.21	0.06	<0.0001	0.06	0.06	0.02	0.020	0.05	-0.11	0.14	0.441	0.05
PA	-0.03	0.13	0.806		-0.03	0.12	0.828		-0.01	0.05	0.828		-0.11	0.30	0.711	
FHH	-0.10	0.13	0.427		-0.08	0.13	0.539		-0.04	0.05	0.539		-0.20	0.30	0.506	
Smoking	-0.33	0.30	0.277		-0.23	0.22	0.301		-0.13	0.11	0.301		-0.75	0.92	0.414	

Data presented as β-coefficient, standard error (SE) and p-value. As well as R^2 for each model. 'Baseline' represents the baseline body fat and fat distribution variable and 'Δ' represents the change in body fat and fat distribution variable; SBP, systolic blood pressure; DBP, diastolic blood pressure; MAP, mean arterial blood pressure; PP, pulse pressure; BMI, body mass index; WC, waist-circumference; HC, hip-circumference, WHtR, waist-to-height-ratio; PA, physical activity; FHH, family history of hypertension.
