# Supplementary material for: The Role of Body Fat and Fat Distribution in Hypertension Risk in Urban Black South African Women
Source: PLoS One. 2016 May 12;11(5):e0154894. doi: 10.1371/journal.pone.0154894 (PMC4865112; doi:10.1371/journal.pone.0154894)
Supplement: S2 Table — Data presented as β-coefficient, standard error (SE) and p-value. As well as R2 for each model. ‘Baseline’ represents the baseline body fat and fat distribution variable and ‘Δ’ represents the change in body fat and fat distribution variable; SBP, systolic blood pressure; DBP, diastolic blood pressure; MAP, mean arterial blood pressure; PP, pulse pressure; FM, fat mass; PA, physical activity; FHH, family history of hypertension. (PDF) [file pone.0154894.s003.pdf]

S2 Table: Regression coefficients for multiple robust linear models for the prediction of blood pressure at follow-up by DXA-derived measures, adjusted for age, baseline and change in body composition, physical activity, family history of hypertension and tobacco use.

DXA-derived measures	SBP				DBP				MAP				PP			
	β	SE	*p*	R^2	β	SE	*p*	R^2	β	SE	*p*	R^2	β	SE	*p*	R^2
**Fat mass**																
Age	0.09	0.08	0.264		-0.03	0.09	0.768		0.03	0.04	0.462		0.38	0.20	0.059	
Baseline FM	0.10	0.09	0.306		0.18	0.07	0.019		0.05	0.04	0.158		0.00	0.23	0.986	
Δ FM	-0.13	0.09	0.143	0.06	0.05	0.08	0.504	0.05	-0.04	0.04	0.326	0.06	-0.49	0.21	0.024	0.08
PA	-0.34	0.17	0.056		-0.25	0.16	0.122		-0.14	0.07	0.054		-0.65	0.38	0.093	
FHH	-0.03	0.17	0.878		0.07	0.16	0.670		0.00	0.07	0.985		-0.30	0.39	0.438	
Smoking	-0.12	0.45	0.792		0.24	0.21	0.238		-0.00	0.16	0.975		-0.95	1.45	0.513	
**Trunk FM**																
Age	0.09	0.08	0.301		-0.03	0.09	0.719		0.02	0.04	0.512		0.37	0.20	0.067	
Baseline TFM	0.18	0.10	0.070		0.23	0.08	0.004		0.09	0.04	0.030		0.15	0.23	0.517	
Δ TFM	-0.07	0.10	0.441	0.07	0.12	0.08	0.170	0.07	-0.01	0.04	0.824	0.07	-0.44	0.22	0.052	0.08
PA	-0.35	0.17	0.058		-0.26	0.16	0.091		-0.15	0.07	0.058		-0.63	0.39	0.111	
FHH	-0.05	0.17	0.771		0.07	0.16	0.665		-0.01	0.07	0.931		-0.37	0.39	0.339	
Smoking	-0.06	0.44	0.886		0.31	0.19	0.114		0.02	0.15	0.891		-0.91	1.47	0.536	
**Arm FM**																
Age	0.09	0.08	0.269		-0.03	0.09	0.719		0.03	0.04	0.478		0.39	0.20	0.060	
Baseline AFM	0.01	0.11	0.942	0.06	0.19	0.09	0.032	0.05	0.03	0.05	0.561	0.05	-0.27	0.28	0.341	0.08
Δ AFM	-0.16	0.08	0.054		0.03	0.07	0.650		-0.05	0.03	0.161		-0.56	0.22	0.010	
PA	-0.35	0.17	0.057		-0.25	0.16	0.114		-0.15	0.07	0.052		-0.66	0.39	0.090	
FHH	-0.00	0.17	0.994		0.08	0.16	0.597		0.01	0.07	0.876		-0.26	0.40	0.518	

Smoking	-0.10	0.43	0.812		0.22	0.22	0.319		-0.00	0.15	0.988		-0.85	1.36	0.533	
**Leg FM**																
Age	0.10	0.08	0.220		-0.02	0.09	0.833		0.03	0.04	0.397		0.40	0.20	0.052	
Baseline LFM	0.02	0.09	0.802		0.08	0.07	0.258		0.02	0.04	0.617		-0.07	0.20	0.726	
Δ LFM	-0.17	0.09	0.047	0.06	-0.03	0.08	0.656	0.03	-0.06	0.04	0.093	0.05	-0.43	0.19	0.025	0.07
PA	-0.36	0.17	0.052		-0.25	0.16	0.119		-0.15	0.07	0.057		-0.70	0.38	0.076	
FHH	-0.01	0.17	0.941		0.09	0.16	0.579		0.02	0.07	0.818		-0.22	0.39	0.567	
Smoking	-0.17	0.45	0.700		0.14	0.24	0.547		-0.04	0.16	0.823		-0.93	1.43	0.516	

Data presented as β-coefficient, standard error (SE) and p-value. As well as R^2 for each model. 'Baseline' represents the baseline body fat and fat distribution variable and 'Δ' represents the change in body fat and fat distribution variable; SBP, systolic blood pressure; DBP, diastolic blood pressure; MAP, mean arterial blood pressure; PP, pulse pressure; FM, fat mass; PA, physical activity; FHH, family history of hypertension.
