# Supplementary material for: The Role of Body Fat and Fat Distribution in Hypertension Risk in Urban Black South African Women
Source: PLoS One. 2016 May 12;11(5):e0154894. doi: 10.1371/journal.pone.0154894 (PMC4865112; doi:10.1371/journal.pone.0154894)
Supplement: S3 Table — Data presented as odds ratio, 95% confidence interval (CI), area under ROC curve (AUC). BP, blood pressure; BC, body composition; Δ BC, change in body composition; PA, physical activity; FHH, family history of hypertension. (PDF) [file pone.0154894.s004.pdf]

S3 Table: Multiple logistic regression analysis of anthropometric-derived measures of body composition as predictors of hypertension, adjusted for age, baseline blood pressure, baseline and change in body composition, physical activity, family history of hypertension and tobacco use.

Anthropometric-derived measures	Odds ratio	95% CI	p	AUC
**BMI**				
Model (n = 330)				
Age	1.54	1.20 – 2.00	0.001	
Baseline BP	2.12	1.61 – 2.7	< 0.0001	
Baseline BMI	1.46	1.13 – 1.90	0.04	
Δ BMI	1.20	0.95 – 1.53	0.134	0.76
PA	1.10	0.66 – 1.82	0.723	
FHH	1.37	0.83 – 2.25	0.213	
Smoking	1.32	0.37 – 4.73	0.667	
**WC**				
Model (n = 330)				
Age	1.49	1.15 – 1.93	0.002	
Baseline BP	2.16	1.64 – 2.85	< 0.0001	
Baseline WC	1.57	1.18 – 2.09	0.002	
Δ WC	1.32	1.03 – 1.70	0.030	0.76
PA	1.09	0.66 – 1.82	0.727	
FHH	1.39	0.84 – 2.83	0.197	
Smoking	1.22	0.34 – 4.43	0.759	
**HC**				
Model (n = 330)				
Age	1.53	1.19 – 1.98	0.001	0.76
Baseline BP	2.12	1.62 – 2.79	< 0.0001	

Baseline HC	1.32	1.02 – 1.71	0.033	
Δ HC	1.41	1.09 – 1.84	0.010	
PA	1.07	0.64 – 1.77	0.801	
FHH	1.40	0.85 – 2.31	0.184	
Smoking	1.20	0.34 – 4.26	0.782	
**WHtR**				
Model (n = 330)				
Age	1.48	1.15 – 1.92	0.003	
Baseline BP	2.17	1.64 – 2.85	< 0.0001	
Baseline WHtR	1.68	1.26 – 2.23	< 0.0001	
Δ WHtR	1.35	1.05 – 1.746	0.021	0.77
PA	1.11	0.66 – 1.85	0.700	
FHH	1.37	0.83 – 2.26	0.214	
Smoking	1.23	0.34 – 4.51	0.752	

Data presented as odds ratio, 95% confidence interval (CI), area under ROC curve (AUC). BP, blood pressure; BC, body composition; Δ BC, change in body composition; PA, physical activity; fam Hx HPT, family history of hypertension.
