# Supplementary material for: The Role of Body Fat and Fat Distribution in Hypertension Risk in Urban Black South African Women
Source: PLoS One. 2016 May 12;11(5):e0154894. doi: 10.1371/journal.pone.0154894 (PMC4865112; doi:10.1371/journal.pone.0154894)
Supplement: S4 Table — Data presented as odds ratio, 95% confidence interval (CI), area under ROC curve (AUC). BP, blood pressure; Δ, change in body composition; FM, fat mass; PA, physical activity; FHH, family history of hypertension. (PDF) [file pone.0154894.s005.pdf]

S4 Table: Multiple logistic regression analysis of DXA-derived measures of body composition as predictors of hypertension, adjusted for age, baseline blood pressure, baseline and change in body composition, physical activity, family history of hypertension and tobacco use.

DXA-derived measures	Odds ratio	95% CI	p	AUC
**Fat mass**				
Model (n = 189)				
Age	1.47	1.06 – 2.04	0.023	
Baseline BP	1.91	1.35 – 2.72	< 0.0001	
Baseline fat mass	1.40	0.97 – 2.02	0.073	0.72
Δ fat mass	1.14	0.79 – 1.65	0.486	
PA	0.87	0.45 – 1.70	0.686	
FHH	1.30	0.68 2.48	0.424	
Smoking	1.77	0.30 – 10.55	0.529	
**Trunk fat mass**				
Model (n = 189)				
Age	1.45	1.05 – 2.02	0.026	
Baseline BP	1.93	1.36 – 2.74	< 0.0001	
Baseline trunk FM	1.51	1.05 – 2.18	0.028	0.73
Δ trunk FM	1.22	0.83 – 1.81	0.314	
PA	0.85	0.43 – 1.66	0.631	
FHH	1.31	0.68 – 2.51	0.414	
Smoking	1.86	0.31 – 11.25	0.499	
**Arm fat mass**				
Model (n = 189)				
Age	1.44	1.03 – 2.00	0.031	
Baseline BP	1.90	1.34 – 2.71	< 0.0001	0.72
Baseline arm FM	1.55	1.00 – 2.38	0.048	
Δ arm FM	1.15	0.78 – 1.72	0.492	

PA	0.88	0.45 – 1.72	0.703	
FHH	1.31	0.69 – 2.51	0.407	
Smoking	1.72	0.29 – 10.23	0.551	
**Leg fat mass**				
Model (n = 189)				
Age	1.48	1.06 – 2.05	0.020	
Baseline BP	1.96	1.38 – 2.79	< 0.0001	
Baseline leg FM	1.16	0.83 – 1.64	0.387	
Δ leg FM	1.02	0.73 – 1.42	0.923	0.72
PA	0.85	0.44 – 1.66	0.636	
FHH	1.30	0.68 – 2.46	0.424	
Smoking	1.49	0.27 – 8.38	0.648	

Data presented as odds ratio, 95% confidence interval (CI), area under ROC curve (AUC). BP, blood pressure; Δ, change in body composition; FM, fat mass; PA, physical activity; FHH, family history of hypertension.
